# Defining and targeting macrophage heterogeneity in the mammary gland and breast cancer

**DOI:** 10.1002/cam4.7053

**Published:** 2024-03-01

**Authors:** Alexis K. Elfstrum, Aditi S. Bapat, Kathryn L. Schwertfeger

**Affiliations:** ^1^ Microbiology, Immunology, and Cancer Biology Graduate Program University of Minnesota Minneapolis Minnesota USA; ^2^ Molecular Pharmacology and Therapeutics Graduate Program University of Minnesota Minneapolis Minnesota USA; ^3^ Department of Laboratory Medicine and Pathology University of Minnesota Minneapolis Minnesota USA; ^4^ Masonic Cancer Center University of Minnesota Minneapolis Minnesota USA; ^5^ Center for Immunology University of Minnesota Minneapolis Minnesota USA

**Keywords:** breast cancer, LYVE‐1, macrophage, mammary gland, TREM2

## Abstract

**Introduction:**

Macrophages are innate immune cells that are associated with extensive phenotypic and functional plasticity and contribute to normal development, tissue homeostasis, and diseases such as cancer. In this review, we discuss the heterogeneity of tissue resident macrophages in the normal mammary gland and tumor‐associated macrophages in breast cancer. Tissue resident macrophages are required for mammary gland development, where they have been implicated in promoting extracellular matrix remodeling, apoptotic clearance, and cellular crosstalk. In the context of cancer, tumor‐associated macrophages are key drivers of growth and metastasis via their ability to promote matrix remodeling, angiogenesis, lymphangiogenesis, and immunosuppression.

**Method:**

We identified and summarized studies in Pubmed that describe the phenotypic and functional heterogeneity of macrophages and the implications of targeting individual subsets, specifically in the context of mammary gland development and breast cancer. We also identified and summarized recent studies using single‐cell RNA sequencing to identify and describe macrophage subsets in human breast cancer samples.

**Results:**

Advances in single‐cell RNA sequencing technologies have yielded nuances in macrophage heterogeneity, with numerous macrophage subsets identified in both the normal mammary gland and breast cancer tissue. Macrophage subsets contribute to mammary gland development and breast cancer progression in differing ways, and emerging studies highlight a role for spatial localization in modulating their phenotype and function.

**Conclusion:**

Understanding macrophage heterogeneity and the unique functions of each subset in both normal mammary gland development and breast cancer progression may lead to more promising targets for the treatment of breast cancer.

## 1 INTRODUCTION

Macrophages are innate immune cells that were first reported at the end of the nineteenth century and were initially defined based on their phagocytic functions.^1^ However, more recent studies have revealed that while phagocytosis remains a key function of macrophages, these cells are involved in numerous additional biological processes that include driving inflammation in response to infiltrating pathogens, maintaining tissue homeostasis, and resolving tissue damage.^2^, ^3^ While initially thought to be derived solely from bone marrow‐derived cells, it is now clear that macrophages can also be derived from embryonic sources.^1^, ^4^ Monocyte‐derived macrophages are recruited into tissues, typically in response to pathogen infiltration or tissue injury. Tissue‐resident macrophages reside within tissues, where they not only respond to pathogens but also contribute to tissue homeostasis and are capable of self‐renewal. Tissue‐resident macrophages are involved in regulating homeostasis in nearly every organ of the body, and their function, phenotype, and effect varies greatly depending on the context.^1^, ^2^, ^5^

In this review, we focus on describing emerging studies that highlight the heterogeneity of macrophages in the normal mammary gland and breast cancer. Macrophages are an integral component of mammary gland development that contribute to extracellular matrix remodeling and apoptotic clearance.^6^, ^7^ These essential homeostatic functions are appropriated in breast cancer to support tumor growth and metastasis. Therefore, understanding the functions of macrophages in the normal mammary gland setting has the potential for providing key insights into macrophage function during tumor progression. In this review, we will discuss macrophage functions in the normal mammary gland and breast tumor, as well as the heterogeneity of macrophages within each setting. In addition, we will highlight two recently discovered macrophage subsets within breast cancer, lymphatic vessel endothelial hyaluronan receptor 1 (LYVE‐1)‐expressing macrophages and lipid‐associated macrophages (LAMs), and discuss emerging efforts to target these macrophages. Understanding the biology and heterogeneity of macrophages within mammary tissue and tumors will ultimately lead to the development of therapeutic approaches that effectively target the tumor‐promoting functions and/or enhance the anti‐tumor functions of this key population of cells within the tumor microenvironment.

## 2 MACROPHAGE HETEROGENEITY IN NORMAL TISSUE

Advances in technologies that allow for analysis of cells at the single cell level have revealed that macrophages represent a phenotypically heterogeneous cell population in normal tissues and in various diseases states. Heterogeneity can be identified in the context of both macrophage identity and activation state. Macrophage identity is determined by a number of factors including ontogeny, exposure to local tissue‐derived factors, and length of time spent in the tissue.^8^ These exposures contribute to the development of distinct subtypes of tissue‐resident macrophages, which are associated with distinct enhancer and super enhancer landscapes that are driven by tissue‐specific factors.^9^ The activation state, or polarization, of macrophages is driven by acute factors that drive transcriptional responses that lead to the rapid ability of macrophages to respond to challenges in their environment. Whereas activation states are generally thought to be plastic and responsive to local changes within the tissue environment, macrophage identity is thought to be more stable.^8^ Thus, when defining macrophage heterogeneity, it is important to consider both identity and activation state, which likely converge to regulate their function.

Although the factors that drive the phenotypic and functional heterogeneity of macrophages are not fully understood, various studies have linked heterogeneity with differences in ontogeny and spatial localization within tissues. Macrophages can be derived from self‐renewing tissue‐resident macrophage populations or recruited as monocytes from the bloodstream, which then enter the tissue and differentiate into macrophages.^1^ Historically thought to have originated exclusively from bone marrow‐derived progenitors, lineage tracing studies in mouse models have demonstrated that tissue‐resident macrophages in some organs are derived from embryonic sources, such as the yolk sac or the fetal liver, and self‐sustain within certain organs through adulthood, such as microglia in the brain and Langerhans cells in the skin.^2^, ^10^, ^11^, ^12^, ^13^, ^14^ Resident macrophages in other sites, such as in the intestine, are seeded early from embryonic sources and are then replaced by bone marrow‐derived macrophages during adulthood.^15^ Macrophages from both embryonically‐derived and bone marrow‐derived lineages generally require the macrophage colony stimulating factor (CSF1R) for differentiation, which binds to ligands CSF‐1 and interleukin (IL)‐34.^16^, ^17^ However, other factors, such as granulocyte macrophage colony stimulating factor (GM‐CSF), are also involved in regulating homeostasis in certain populations of macrophages, such as alveolar macrophages.^18^, ^19^, ^20^

Tissue‐resident macrophages contribute to immune surveillance and phagocytosis of apoptotic debris and have various tissue‐specific functions.^2^ For example, alveolar macrophages in the lung are involved in surfactant uptake, clearance, and gas exchange.^21^ Kupffer cells in the liver degrade toxins from sinusoidal blood and clear senescent erythrocytes.^22^ Microglia in the brain contribute to the pruning of synapses and regulation of neuronal circuits.^23^ Langerhans cells serve as initial responders to invading pathogens in the epidermis and have potent antigen‐presentation capabilities.^24^ Several studies have revealed that the functions of these tissue‐resident macrophage function are driven by key transcriptional and epigenetic regulators that are modulated by tissue‐specific factors. Pioneering work in this field focused on regulation of peritoneal macrophages and demonstrated that locally derived retinoic acid from the omentum induces expression of the transcription factor GATA binding protein 6 (GATA‐6), which regulates the expression of key genes involved in peritoneal macrophage function.^25^ Peroxisome proliferator‐activated receptor γ (PPARγ) is critical for modulating GM‐CSF‐mediated differentiation of alveolar macrophages and drives expression of additional transcription factors important for alveolar macrophage function.^26^ Additional work has demonstrated that the tissue environment converges on epigenetics to regulate access to tissue‐specific enhancers in macrophages.^27^ This regulation involves interactions between PU.1, which is a lineage‐determining transcription factor in macrophages, and tissue‐specific transcription factors.^9^ Findings such as these highlight the complex mechanisms involved in driving tissue‐specific macrophage function.

To highlight the role of the microanatomical niche in driving macrophage heterogeneity, studies by Chakarov et al.^28^ demonstrated the presence of two distinct tissue resident populations that differentiate from lymphocyte antigen 6 complex, locus C high (Ly6C^hi^) blood‐derived monocytes, but exhibit distinct phenotypes depending on their localization within the tissue. Specifically, macrophages that are localized to nerves express high levels of major histocompatibility complex II (MHCII), and macrophages that are localized to blood vessels are associated with high levels of the cell surface marker LYVE‐1.^28^ Thus, in addition to transcriptional and epigenetic regulation of macrophages, localization within distinct microanatomical niches may also contribute to tissue‐resident macrophage phenotype.

Although some tissue‐resident macrophages have clearly defined functions within the tissues where they reside, recent studies have addressed the possibility that there are subsets of tissue‐resident macrophages with conserved functions across tissues. Using single cell RNA sequencing (scRNA‐seq) approaches, Dick et al.^5^ defined three distinct subsets of macrophages based on expression of the markers T cell immunoglobulin and mucin domain containing 4 (TIM4), LYVE‐1, folate receptor beta (FOLR2), chemokine receptor C‐C motif chemokine receptor 2 (CCR2), and major histocompatibility complex II (MHC II). Specifically, they identified a subset of macrophages that expresses the cell surface markers TIM4, LYVE‐1, and FOLR2 that is defined as TLF+ macrophages, which are highly conserved across multiple tissues including heart, liver, lung, kidney, brain, and adipose. Two additional subsets are defined as CCR2+ and MHCII^hi^. While both of these populations express MHCII, the MHCII^hi^ population is notable for its lack of CCR2 expression. Lineage tracing studies demonstrate differences in the ontogenies of these macrophage subsets. Specifically, the TLF+ population shows slow turnover rates and the least dependence on monocyte recruitment, whereas the CCR2+ macrophages exhibit the highest rate of monocyte‐dependent turnover. These studies highlight the heterogeneity in macrophages that exist in normal tissues prior to the onset of disease and raise the possibility that cancer cells may be capable of recruiting and leveraging these tissue‐resident macrophage populations as they establish and grow within tissues.

## 3 MACROPHAGE FUNCTION AND HETEROGENEITY IN NORMAL MAMMARY TISSUE

The mammary gland is a dynamic tissue that undergoes continual modification throughout embryonic development, puberty, pregnancy, lactation, and involution.^29^ There are five pairs of mammary glands in the mouse that are composed of epithelial structures embedded within an adipose‐rich fat pad. Mammary gland development begins during embryogenesis, during which time a rudimentary ductal structure is formed.^29^ During puberty, the epithelial duct elongates through the mammary fat pad, which is driven by highly proliferative structures referred to as terminal end buds. Once established, the ductal tree undergoes estrous cycle‐related epithelial budding and regression but otherwise remains relatively quiescent until pregnancy. The epithelium undergoes extensive lobuloalveolar development during pregnancy in preparation for milk production during lactation. Following weaning, the mammary gland is dramatically remodeled during the process of involution, which involves apoptosis of the epithelial cells and extensive stromal remodeling. In addition to epithelial cells and adipocytes, the mammary gland also contains fibroblasts, endothelial cells, lymphatic endothelial cells, and various immune cell populations including T cells, eosinophils, and tissue resident.macrophages.^30^, ^31^, ^32^

Macrophages have been identified in the embryonic mammary gland and have been localized near, but not in direct contact with, the mammary epithelial buds at E14.5.^33^ Ductal elongation during pubertal mammary gland development involves extensive interactions between the epithelial cells of the ducts and the surrounding cells of the stroma, governed by cytokines, chemokines, growth factors, and hormones.^34^, ^35^, ^36^ Macrophages have been found near terminal end buds during ductal elongation, where they contribute to ductal elongation and collagen fibrillogenesis around the terminal end bud, and have also been proposed to phagocytose apoptotic epithelial cells.^37^, ^38^, ^39^ Recent studies have also demonstrated that ductal elongation is modulated by atypical chemokine receptor (ACKR2) as well as C‐C Motif Chemokine Ligand 7 (CCL7), which mediates the recruitment of macrophages to the ductal epithelium.^40^ These macrophages express C‐C Motif Chemokine Receptor 1 (CCR1), which is upregulated by estrogen, a hormone that is critical for driving ductal elongation.

**FIGURE 1:**
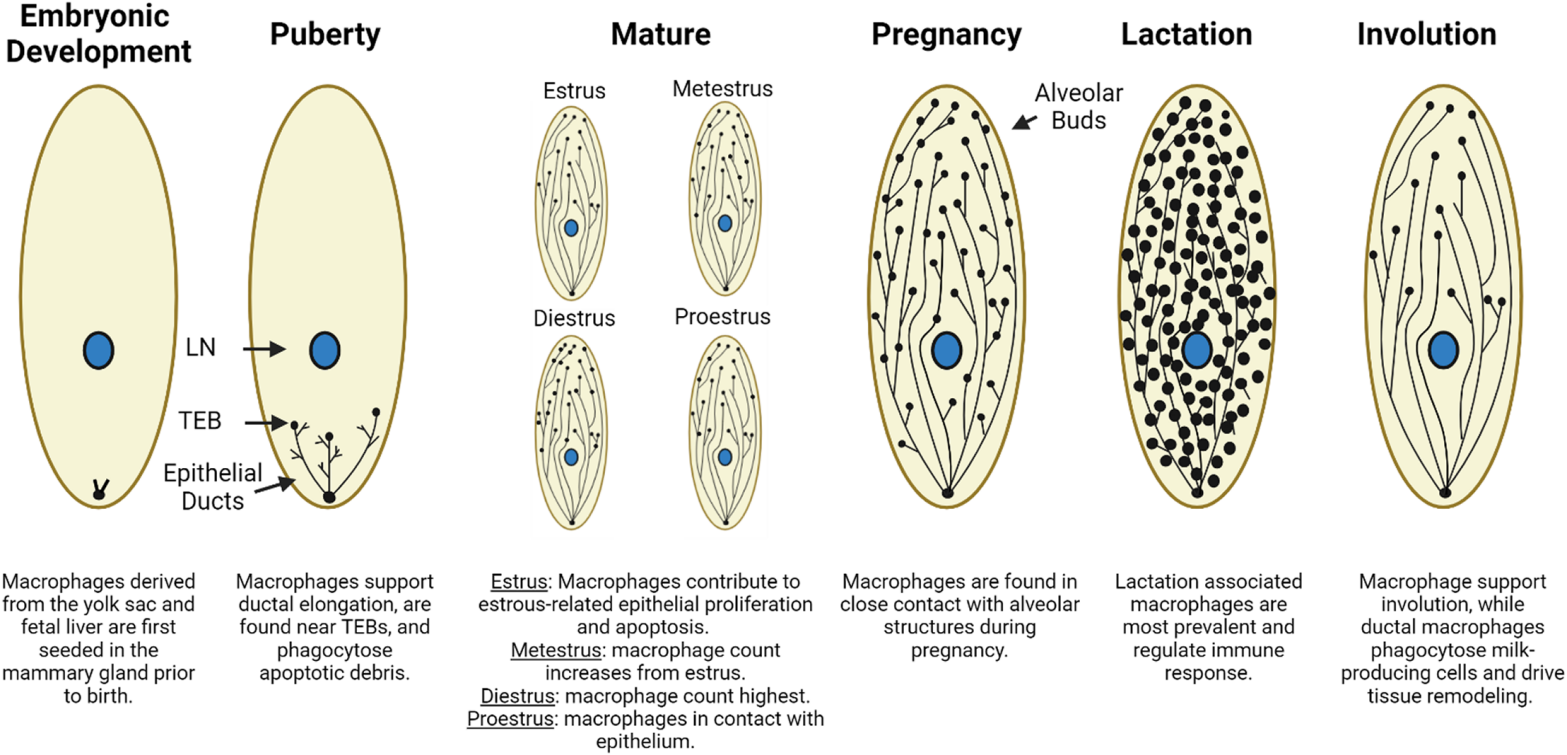
Macrophages support mammary gland development. Macrophages first seed the mammary gland during embryonic development and later support development through puberty, the estrous cycle, pregnancy, lactation, and involution.^39^, ^40^, ^41^, ^42^, ^43^, ^44^ LN: lymph node, TEB: terminal end bud. Created using Biorender.com.

In addition to ductal elongation, tissue‐resident macrophages have been shown to contribute to other stages of mammary gland development including estrous cycle‐related epithelial remodeling, pregnancy, and involution (Figure 1). Analysis of the mammary gland during the estrous cycle demonstrated that macrophages are found in greatest abundance at diestrus and that macrophages are in contact with ductal epithelium peak at the proestrus stage.^39^ Furthermore, macrophage depletion studies demonstrated that these macrophages contribute to estrous‐related epithelial proliferation and apoptosis.^39^ Macrophages are found in close contact with alveolar structures during pregnancy, and macrophage deficiency is associated with reduced alveolar development during pregnancy.^41^, ^42^ A population of monocyte‐derived macrophages associated with lactation has recently been identified both in the tissue and in milk, termed lactation‐induced macrophages (liMacs), which are thought to be involved in immune response.^43^ Finally, macrophages are present during involution, and depletion of macrophages was found to be associated with delayed involution and a reduction in apoptotic epithelial cells, suggesting that macrophages are important during the post‐lactational involution process.^44^

As described above, resident macrophage function is driven by transcriptional and epigenetic mechanisms. While limited studies have been performed to identify specific transcription factors that drive resident macrophage function in the mammary gland, we have previously demonstrated that signal transducer and activator of transcription 5 (STAT5) is selectively expressed in a subset of macrophages in the mammary gland.^45^ Furthermore, genetic deletion of STAT5 in macrophages results in altered ductal development, with reduced ductal elongation, increased branching, and increased epithelial proliferation.^45^ Given the well‐established importance of transcription factors in driving tissue‐specific functions in other organ sites, further studies are needed to identify the key transcriptional and epigenetic factors regulating resident macrophage function in the mammary gland.

Recent studies have used lineage‐tracing models to assess macrophage ontogeny in the mammary gland. Macrophages derived from the yolk sac and fetal liver are first seeded in the mammary gland prior to birth. In adulthood, these tissue resident macrophages outnumber monocyte‐derived macrophages and are marked by cluster of differentiation 206 (CD206), a marker of tissue‐resident macrophages.^46^ Further studies demonstrate that CCR2−/− mice exhibit no change in ductal elongation or branching, suggesting the importance of locally derived macrophages, rather than bone marrow‐derived macrophages, on ductal development.^46^

**FIGURE 2:**
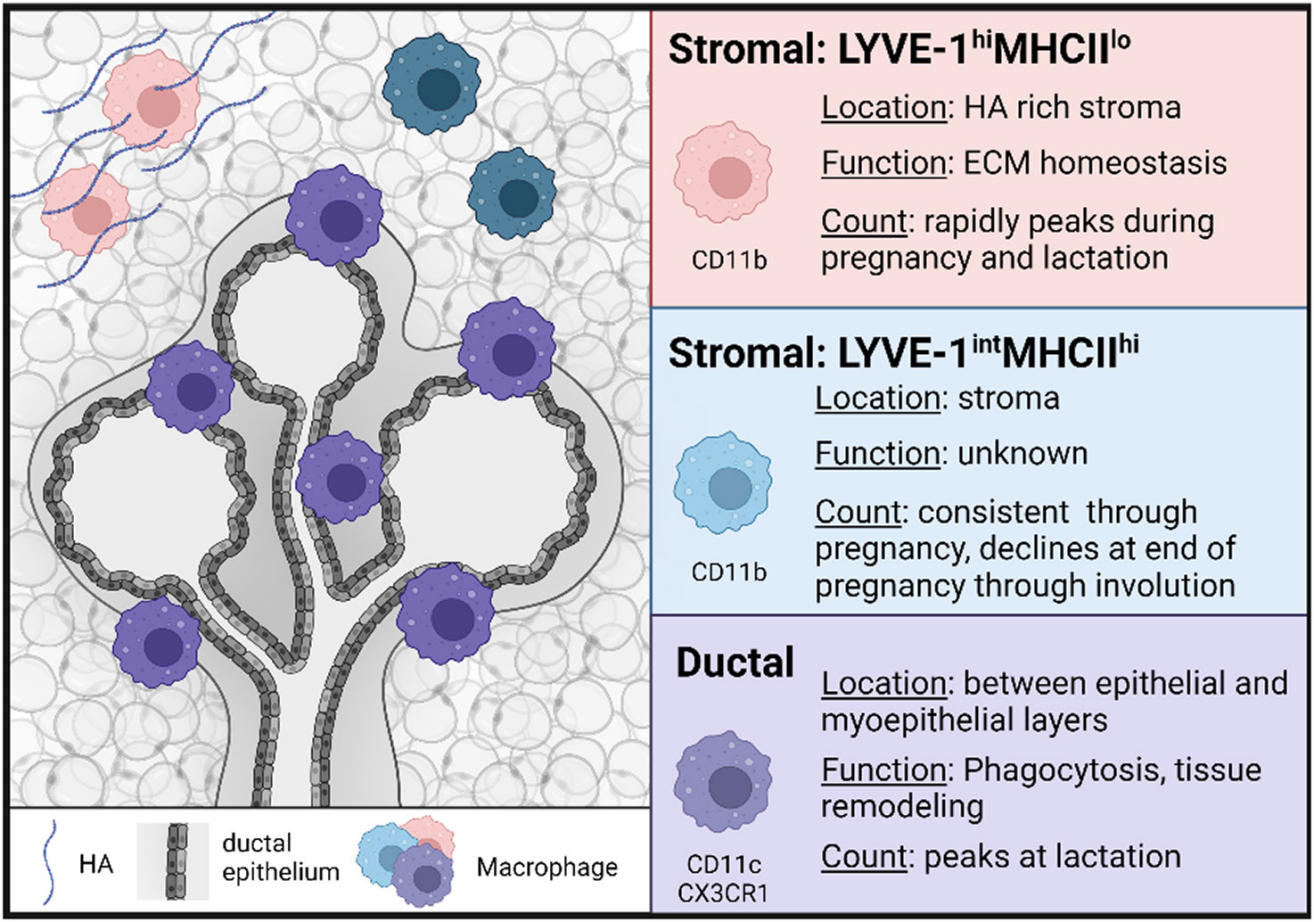
Three macrophage subsets are found in the mouse normal mammary gland. Ductal macrophages are found between the epithelial and myoepithelial layers (purple), while stromal macrophages are found in the adipose stroma of the mammary gland. Stromal macrophages are further divided into LYVE‐1^hi^MHCII^lo^ macrophages (red) and LYVE‐1^int^MHCII^hi^ macrophage (blue).^6^, ^7^, ^43^ Created using Biorender.com.

While the presence and importance of macrophages in various stages of mammary gland development have been documented, recent studies have begun to highlight the extent of heterogeneity of tissue‐resident macrophages in the mammary gland. Studies by Dawson et al.^7^ identified three distinct populations of macrophages within mammary tissue including a ductal population and two stromal populations of macrophages (Figure 2). Ductal macrophages, which express cluster of differentiation 11c (CD11c) and C‐X3‐C motif chemokine receptor 1 (CX3CR1), are found in direct contact with epithelial ducts and located between the epithelial and closely associated myoepithelial layers of cells. Ductal‐associated macrophages are also referred to as liMacs, as their abundance peaks during lactation. Although these macrophages were found to have an embryonic origin, they rely on blood‐derived monocytes for expansion in adulthood. Ductal macrophages are found in low abundance in adulthood but are rapidly expanded during pregnancy and lactation.^7^ Following lactation, ductal macrophages phagocytose milk‐producing cells and drive tissue remodeling during involution. Ductal macrophages were also found to have a role in surveying for tissue damage. When the epithelium is damaged, ductal macrophages migrate towards the injury, and 70% of apoptotic epithelial cells were found in contact with ductal macrophages.^7^

In addition to the MHCIIhi ductal‐associated macrophages, two populations of stromal macrophages are found in the mammary stroma, both of which express CD11b and are differentiated by either high or low expression of MHCII7. Dawson et al. demonstrated that MHCIIlo macrophages express high levels of LYVE‐1, while MHCIIhi macrophages express intermediate levels of LYVE‐15. Cansever et al. reported an F4/80hi and MHCIIlo stromal macrophage population expressing Lyve1 in virgin and lactating mice, in addition to an F4/80lo and MHCIIhi stromal macrophage population that is negative for Lyve143. Further, in lactating mammary glands, the F4/80hi subset expresses Folr2, Lyve1, and Timd4, while the F4/80lo subset has lower expression of these genes. Finally, we have also demonstrated F4/80hi coexpression with LYVE‐1 in nulliparous mammary glands.^6^ Collectively these studies highlight the heterogeneity of tissue‐resident macrophages in the mammary gland. Further analysis of the spatial localization of macrophages during each distinct stage of development will provide additional insights into how ductal and stromal macrophages respond to the dramatic changes that occur during mammary gland development.

While the MHCIIhi stromal macrophages have not yet been studied in detail, studies by us and others have examined the localization and function of the LYVE‐1 expressing stromal macrophages. LYVE‐1 is a receptor for the extracellular matrix molecule hyaluronan.^6^, ^32^ We have recently demonstrated that hyaluronan is localized throughout the adipose stroma and adjacent to epithelial ducts in the developing mammary gland, as well as during pregnancy and involution.^47^ Consistent with the ability of LYVE‐1 to bind hyaluronan, LYVE‐1+ macrophages are found in close proximity to hyaluronan‐enriched septa within the mammary gland, and depletion of macrophages results in increased hyaluronan accumulation.^6^ Furthermore, analysis of RNA sequencing of LYVE‐1+ macrophages from the mammary gland suggests that these macrophages are associated with extracellular matrix remodeling pathways. Together, these findings suggest that LYVE‐1+ macrophages are associated with maintaining extracellular matrix homeostasis in the adipose stroma of the mammary gland.

Additional studies have demonstrated the presence of LYVE‐1+ macrophages in both the nulliparous mammary gland and during mammary gland involution. LYVE‐1+ macrophages are found in low abundance in virgin mammary tissue and rapidly rise during pregnancy and lactation before regressing during involution.^43^ LYVE‐1+ macrophages are present during involution, where they have been shown to incorporate into lymphatic vessels, following which they decrease during the resolution of involution.^48^ The frequency of these macrophages increases during involution, peaking at involution day 6 and regressing thereafter.^48^ Using flow cytometry, Wilson et al.^49^ characterized changes in multiple macrophage subsets throughout pubertal development, pregnancy, lactation, and involution in the mammary gland, validating the diversity and dynamics of macrophages throughout mammary gland development. While ductal macrophages were found to peak in abundance during lactation and early involution, macrophages expressing CD206, a protein also expressed on LYVE‐1+ macrophages, peak in abundance at involution day 5.^6^, ^49^

Macrophage subsets within the mammary gland have distinct roles during mammary gland development and are localized to specific tissue regions. While the functions of these macrophage subsets are essential for the normal mammary gland, their presence and localization within both epithelial and stromal compartments raise the possibility that their functions can be leveraged during mammary tumorigenesis. Macrophage‐mediated tissue remodeling and apoptotic clearance, while essential for ductal elongation, also have the potential to support mammary tumor growth.^50^ Further, macrophages with ductal and stromal macrophage gene signatures have been identified in mammary tumors.^7^ Understanding macrophage function in the normal mammary gland is essential for understanding, and ultimately targeting, macrophages within tumors.

## 4 MACROPHAGES IN BREAST CANCER

Given the localization and importance of macrophages in the normal mammary gland, it is not surprising that macrophages have also been identified in breast cancer. Breast cancer is a complex disease with distinct molecular subtypes.^51^, ^52^ The luminal A subtype is hormone receptor (HR) positive and can express either estrogen receptor (ER) or progesterone receptor (PR), and is negative for human epidermal growth factor receptor 2 (HER2). The luminal B subtype is HR+ and HER2+/−. The HER2 subtype expresses the HER2 protein, while the basal‐like subtype is negative for ER, PR, and HER2, which corresponds with the clinical triple‐negative breast cancer (TNBC) subtype. Macrophages have been identified in human (patient) breast cancer samples across subtypes using immunohistochemistry‐based approaches. Luminal A breast cancer has significantly lower infiltration of CD68+ macrophages than luminal B, HER2+, and TNBC subtypes, with the latter three subtypes displaying relatively similar macrophage counts.^53^ Increased CD68+ macrophage infiltration is positively correlated with high histological grade and high Ki67 regardless of hormone receptor status.^53^ Another study, which differentiated between macrophages in the stroma and the tumor parenchyma, found that CD163+ macrophage infiltration in the breast tumor stroma correlates with larger tumors, proliferation, and hormone receptor‐negative status. However, this trend does hold for intratumoral macrophage infiltration.^54^ Additionally, total CD68+ macrophage count has been positively associated with hormone receptor negative status and TNBC.^55^, ^56^ In patients with invasive ductal carcinoma, the scavenger receptor CD204 is associated with poor prognosis.^57^ These studies demonstrate the presence of macrophages across breast cancer subtypes. However, studies have shown varying degrees of expression of the surface receptors CD68 and CD163, utilized by these studies in their quantification of tumor‐associated macrophages in non‐macrophage cell types such as monocytes, dendritic cells, and neutrophils as well as in non‐immune cells.^58^, ^59^, ^60^, ^61^ Specificity of macrophage markers thus needs to be considered for studies correlating macrophage expression and patient survival and prognosis.

While macrophages have long been known to be present within the tumor microenvironment, their specific function in the context of tumor growth and progression was unclear before pioneering work performed by Dr. Jeffrey Pollard and colleagues demonstrated the importance of macrophages during tumor metastasis. Using the mouse mammary tumor virus (MMTV)‐polyoma middle T (PyMT) mammary tumor model, this group demonstrated that macrophage deficiency inhibits the ability of tumor cells to metastasize to the lung. Further work demonstrated the presence of a paracrine loop in which breast cancer cells recruit macrophages by producing colony stimulating factor‐1 (CSF‐1), and macrophages promote tumor cell invasiveness by producing epidermal growth factor (EGF)^62^, ^63^, ^64^ These studies set the stage for extensive additional work on the variety of functions of macrophages in the growth, metastasis, and response to therapy in breast and other tumor types.^65^, ^66^, ^67^, ^68^, ^69^, ^70^

Macrophages are now well‐established components of the tumor microenvironment and are known to be involved from the early stages of cancer initiation to malignancy and metastasis.^71^, ^72^ Several studies have shown that macrophage infiltration within the tumor correlates with poor prognosis in breast cancer patients.^73^ A study by Mahmoud et al.^55^ showed shorter disease‐free survival correlated with infiltration of CD68 macrophage populations even though macrophage count was not determined to be an independent prognostic marker. The number of infiltrating macrophages was also associated with a higher risk of distant metastasis in TNBC, coupled with lower overall survival.^54^, ^74^, ^75^ These studies highlight the impact of the tumor‐promoting functions of macrophages on disease progression and patient survival, warranting investigations into their mechanisms within the tumor microenvironment.

## 5 TUMOR ASSOCIATED MACROPHAGE HETEROGENEITY: THE M1/M2 PARADIGM

The various roles of macrophages during the wound healing process highlight their functional and context‐dependent plasticity and have provided key insights into their functions in cancer.^76^ The process of wound healing involves several distinct stages, including hemostasis, inflammation, proliferation, and remodeling.^77^ During the inflammation stage, macrophages serve a pro‐inflammatory function where they are involved in recruiting immune cells, killing pathogens, and presenting antigen to infiltrating T cells.^77^ These macrophages are typically referred to as exhibiting a pro‐inflammatory, or M1, phenotype. M1 macrophages were reported to have a pro‐inflammatory phenotype, with high expression of interleukin (IL)‐1β, interleukin (IL)‐6, and tumor necrosis factor (TNF)‐α.^78^ These macrophages also have the ability to kill pathogens via the production of reactive oxygen species (ROS) and engage adaptive immune responses via antigen presentation.^79^ Macrophages are involved in the proliferation and remodeling phases of wound healing by producing factors that drive fibroblast activation, angiogenesis, epithelial proliferation, extracellular matrix remodeling, and suppression of adaptive immune responses.^80^ This is associated with an anti‐inflammatory, or M2, phenotype.^80^ The plasticity of macrophage function is highly dependent upon the signals they receive within their immediate environment and provides a source for the robust phenotypic heterogeneity observed in both resident and infiltrating macrophages.

This paradigm was adapted to the tumor‐associated macrophage (TAM) field to describe the heterogeneity in phenotype and function observed in macrophages associated with tumors.^81^ M1 macrophages, which express high levels of pro‐inflammatory cytokines and MHCII are referred to as anti‐tumor, whereas M2 macrophages, which express high levels of immunosuppressive factors such as IL‐10 and transforming growth factor (TGF)‐β are referred to as pro‐tumorigenic.^82^ While more recent technological advances have shed additional light on the extent of macrophage heterogeneity in breast cancer, as described in more detail below, the study of TAMs within the context of the M1/M2 paradigm has led to several consistent themes related to TAM function in the tumor microenvironment. These include the regulation of angiogenesis, extracellular matrix remodeling, and immunosuppression. These have been reviewed extensively^80^, ^82^, ^83^, ^84^, ^85^ and will only be briefly summarized here.

Macrophages are well‐known mediators of angiogenesis during wound healing. Within tumors, TAMs contribute to neovascularization by either directly producing vascular endothelial growth factor (VEGF) or indirectly by stimulating vascular endothelial cells to increase VEGF expression, thereby promoting angiogenesis.^86^ TAMs are also recruited to the tumor core in response to hypoxia, which further enhances their pro‐angiogenic functions by inducing VEGF expression.^87^ Hypoxia induces the expression of angiopoietin 2 (ANG2) and its cognate receptor TEK receptor tyrosine kinase (TIE2), increasing TAM migration and activation of vascular endothelial cells.^88^ High levels of macrophages with increased expression of angiogenesis‐promoting genes are also associated with poor prognosis in multiple cancer types.^89^

During tissue remodeling, macrophages are known to produce proteases that breakdown the extracellular matrix (ECM).^83^ In tumors, the production of matrix metalloproteases (MMPs) and cathepsins by TAMs facilitated ECM degradation, which contributes to tumor cell migration and invasion by releasing sequestered tumor‐promoting factors from the matrix and creating space through which the tumor cells are able to invade.^71^, ^90^ Proteases can also contribute to therapeutic resistance. For example, the production of cathepsins B and S by macrophages has been reported to increase the production of tumor‐protective factors in response to chemotherapy.^91^

During the last stage of wound healing, macrophages produce immunosuppressive factors that reduce inflammation and limit tissue damage. Immunosuppression has emerged as a critical function for TAMs in the tumor microenvironment, and TAMs are known to express several factors that inhibit adaptive immune cell function in the tumor microenvironment.^71^ TAMs express various cytokines and chemokines that regulate the recruitment and activation of immune cells to the tumor. IL‐10 and TGF‐β are well‐established immunosuppressive cytokines that can promote the induction of regulatory T cells (Tregs) and the expression of cytotoxic T‐lymphocyte‐associated protein 4 (CTLA4) and forkhead box protein 3 (FoxP3) in CD4+ T cells, both markers of Treg expression.^92^, ^93^ These cytokines can also suppress anti‐tumor T cell activation. TAM‐produced chemokines can also lead to the recruitment of suppressive immune cells to the TME. Increased migration of chemokine receptor 4 (CCR4)‐expressing Tregs in response to chemokines such as CCL22 and CCL17 has been reported in breast and other cancers, including ovarian and gastric tumors.^94^, ^95^, ^96^, ^97^ Finally, TAMs express ligands for inhibitory receptors involved in induced death of effector immune cells such as programmed cell death protein 1 (PD‐1) and cytotoxic T‐lymphocyte antigen 4 (CTLA‐4), directly inhibiting signaling downstream of T cell and B cell receptors.^71^ Activated lymphocytes were shown to increase PD‐L1 and PD‐L2 expression in tumor‐associated macrophages via GM‐CSF and IFN‐γ, suggesting the creation of a feedback loop involving macrophages to dampen anti‐tumor immunity.^98^

While many studies have focused on M2‐like properties of macrophages in tumor progression, M1‐associated factors have also been found to have tumor‐promoting properties. Several cytokines produced by macrophages, such as interleukin‐6 (IL‐6) and cyclooxygenase‐2 (COX‐2), have been implicated in the activation of both pro‐survival and anti‐apoptotic pathways in tumor cells and mediating resistance to chemotherapeutic drugs.^99^, ^100^ Macrophage‐derived COX‐2 has been shown to promote tumor growth in mammary tumor models by a positive feedback loop that activates pro‐survival pathways in tumor cells as well as contributes to therapeutic resistance.^101^, ^102^ Macrophage‐derived IL‐6 activates the STAT3 and Hedgehog pathway in tumor cells, promoting proliferation and chemoresistance.^103^, ^104^ Soluble pro‐inflammatory factors such as CCL2, CCL5, and CCL18 secreted by TAMs from the primary tumor have been implicated in the formation of a pre‐metastatic niche in distant organs such as the lungs and the bones, increasing tumor cell colonization.^105^, ^106^, ^107^ Furthermore, TAM production of CCL2 as a driver of additional macrophage recruitment has also been well documented, and elevated expression of CCL2 is inversely proportional to patient prognosis in breast and ovarian carcinomas.^97^

In contrast, other studies have demonstrated that promoting an M1‐like phenotype is associated with anti‐tumor function. In a study by Tkach et al.,^108^ extracellular vesicles from tumor cells were shown to include an anti‐tumor gene expression signature in TAMs, correlating with better patient outcomes and survival. These macrophages can also express IFN‐related genes such as CXCL10, ISG15, and PDL1 and are similar to M1‐like macrophages within the tumor.^109^, ^110^, ^111^ In addition, we have demonstrated that activation of STAT5, which is a transcription factor that drives M1‐like responses in macrophages, promotes anti‐tumor TAM functions in mammary tumor models.^112^ Thus, M1‐like macrophages likely represent a mix of macrophages with both anti‐ and pro‐tumorigenic functions. Taken together, these studies demonstrate the extensive heterogeneity in macrophage function within the tumor microenvironment.

## 6 MACROPHAGE HETEROGENEITY IN BREAST CANCER: BEYOND M1/M2

**FIGURE 3:**
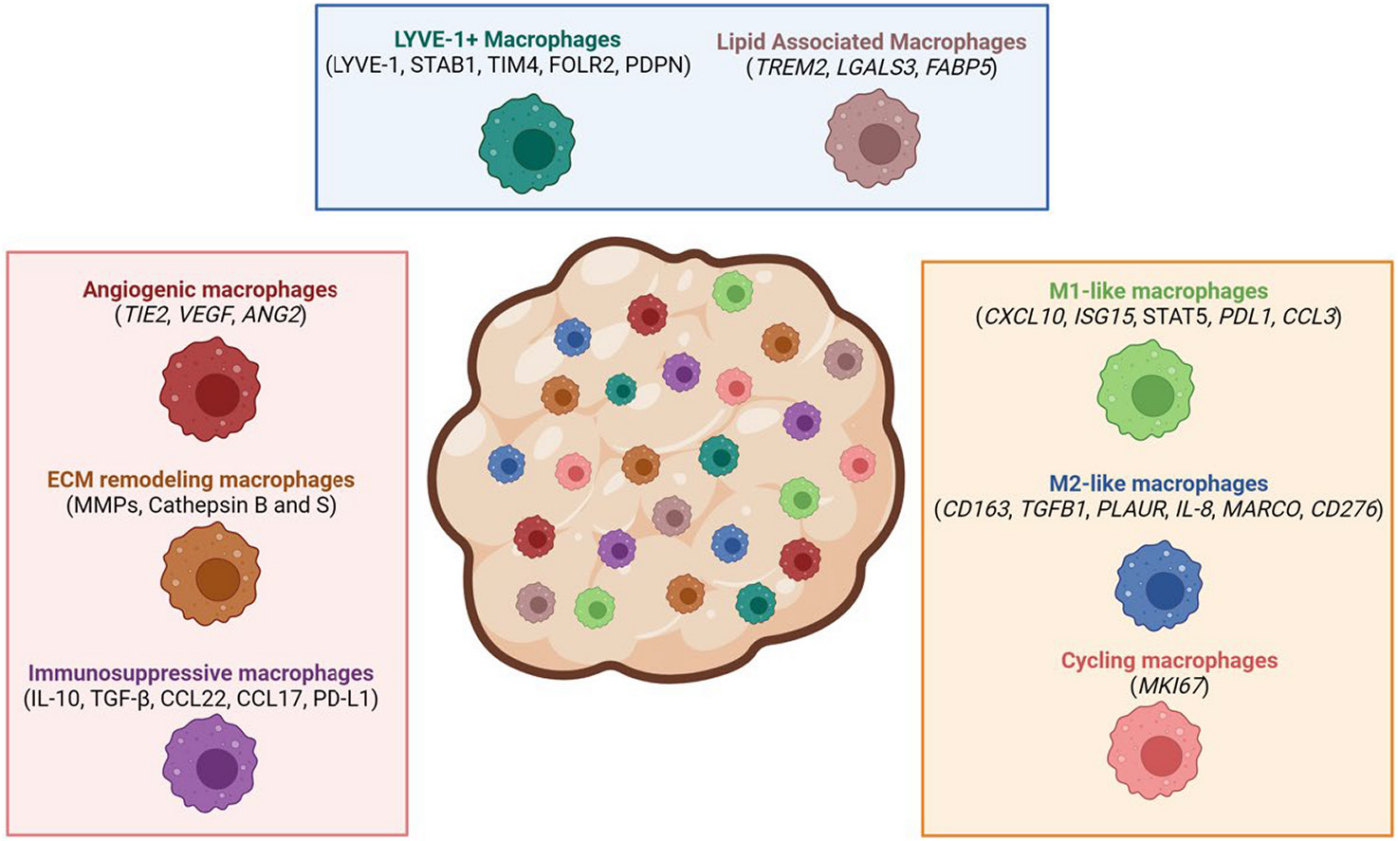
Single‐cell transcriptomic studies have identified multiple macrophage subsets in breast cancer. Recent advances in single‐cell sequences have identified several subsets of macrophages with distinct as well as overlapping markers. The angiogenic, ECM remodeling and immunosuppressive macrophages have been well established and identified in multiple tumor types (pink inset). Transcriptomic studies with human breast cancer samples have further stratified macrophage populations into M1‐like, M2‐like, and cycling (yellow subset). Finally, LYVE‐1+ and lipid‐associated macrophage subsets have been identified recently (blue subset) and are now being investigated as potential targets in solid tumors. Created using Biorender.com.

While it has long been acknowledged that macrophages exist in a spectrum rather than an extreme state of polarization, it was not until technological advances in single‐cell sequencing and subsequent bioinformatic analyses have significantly enhanced our understanding of cellular heterogeneity in tumors. These studies unearthed several distinct subsets of macrophages within the TME with a spectrum of functions that span phenotypes associated with anti‐tumor and tumor‐promoting functions.^82^ Multiple single‐cell sequencing studies in human breast cancer patient samples have identified both known and novel subsets of TAMs (Figure 3).^82^, ^99^

Early scRNA‐seq studies performed with 11 breast cancer patient samples confirmed the presence of M2‐like macrophages within tumors that express markers such as *CD163*, *TGFBI*, *PLAUR* and *IL‐8*.^113^ Another study, which focused specifically on CD45+ cells isolated from 8 primary breast cancers, demonstrated additional heterogeneity with the identification of distinct macrophage subsets that express markers associated with M1‐like (*CCL3*) and M2‐like (*MARCO*, *CD276*) macrophages.^114^ Additional genes that were associated with the M2‐like macrophages include *APOE*, *TREM2*, and *CHIT1*. Another study in which scRNA‐seq was performed on normal, preneoplastic, and invasive breast cancer demonstrated the presence of cycling macrophages that express *MKI67*.^115^ Interestingly, proliferating macrophages have been previously associated with higher grades, poor clinical outcomes, increased aggressiveness, and poor survival.^56^, ^116^, ^117^ Further analysis on this particular scRNA‐seq dataset revealed substantial additional macrophage heterogeneity within both tissue‐resident macrophages and TAMs.^118^ Interestingly, increased levels of tissue‐resident macrophage populations were found to be associated with higher levels of CD8 T cells, NK cells, and dendritic cells, whereas TAMs were associated with immunosuppressive cells. This finding supports previously published work by Nalio Ramos et al.,^119^ in which they demonstrated that macrophages expressing the resident macrophage marker FOLR2 positively correlate with CD8+ T cells, B cells, dendritic cells, and tertiary lymphoid structures, and that this correlates with better outcome in breast cancer patients. Together, these findings suggest that macrophage subsets are associated with distinct immune environments.

The scRNA‐seq analysis of 26 primary breast cancer samples identified several macrophage clusters associated with M1‐like markers (*CXCL10*), two distinct clusters associated with M2‐like markers (*EGR1* and *SIGLEC1*), and two distinct clusters associated with lipid markers (*FABP5* and *APOE*). Furthermore, both the M1‐like and LAM clusters were associated with higher levels of PD‐L1 and PD‐L2, suggesting a link between these subsets and an immunosuppressive environment.^120^ However, it is important to note that it can be challenging to define function of macrophage clusters solely through marker analysis. For example, it is worth noting that SIGLEC1 expression can be induced by IFN‐γ in patients with congenital heart block, sclerosis, and certain viral infections, suggesting an M1‐like phenotype and a tissue‐specific function of the receptor.^121^, ^122^, ^123^ Thus, while identification of clusters is an important first step in characterizing macrophage phenotype, functional studies are required to fully define whether key cluster markers contribute to macrophage function.

A recent study performed scRNA‐seq of samples isolated from the tumor, interface, and normal zones from invasive breast cancers from three patients. M2‐like macrophages that express high levels of the scavenger receptor *SCARA3* and reduced levels of genes associated with an IFN response genes and ROS were found across all three zones. Macrophages associated with high levels of genes associated with IFN and ROS were found in both the tumor and interface zones, whereas macrophages associated with VEGFA expression and a hypoxia‐associated gene expression signature were found only in the tumor zone.^124^ These findings suggest a correlation between spatial localization and macrophage phenotype.

In a study by Onkar et al.,^125^ scRNA‐seq was performed to identify the immune landscapes in human ER‐positive invasive ductal (IDC) and invasive lobular carcinomas (ILC). Macrophages were found to be the predominant immune cell type in both IDC and ILC. Analysis of clusters demonstrated the presence of cycling macrophages (*MKI67*) in both IDC and ILC. However, comparison of macrophages revealed transcriptional diversity between IDC and ILC samples. For example, macrophages from IDC samples were enriched in genes and pathways associated with migration, cell adhesion, activation, and cytotoxicity, whereas macrophages from ILC were found to be enriched in genes associated with regulation of apoptosis, suppression of inflammation as well as T cell‐associated pathways, suggesting potential interactions between macrophages and T cells in ILC. Further analysis also demonstrated that ILC samples were associated with a higher M2/M1 ratio than IDC.

Taken together, these studies demonstrate the presence of heterogeneous subsets of macrophages in human breast cancer samples along with the identification of several new subsets including those associated with tissue resident markers and LAM subsets, which will be described in more detail below. Interestingly, many of these populations have been recently identified in scRNA‐seq analysis of the normal human breast including M1‐like (*IL1B*), M2‐like (*MRC1*), IFN‐associated (*IFIT1*, *IFIT2*), and lipid‐associated (*LPL*) macrophages,^126^ suggesting a link between tissue‐resident macrophages in the normal mammary gland and in tumors. In contrast to mouse models in which lineage tracing methods are available to help define how ontogeny contributes to macrophage identity, less is known about how macrophage identity may contribute to heterogeneity in human samples. Inclusion of additional single cell‐based analysis, such as scATAC‐seq, may help to define enhancer landscapes and identify how human macrophages are regulated at the level of chromatin organization, enhancing our understanding of how tissue resident macrophage identity may contribute to the heterogeneity of human tumor‐associated macrophages. Understanding how these resident macrophages are altered in the context of tumor initiation, growth, and progression will continue to provide key insights into the regulation and functions of these cells in breast cancer.

## 7 EMERGING MACROPHAGE SUBSETS IN THE TUMOR MICROENVIRONMENT

### 7.1 LYVE‐1+ Macrophages

Lymphatic vessel endothelial hyaluronan receptor‐1 was first identified as a marker of lymphatic endothelial cells, however, its expression has also been found in a subset of macrophages found in an array of tissues. First identified in 2006, LYVE‐1+ macrophages were found in a B16F1 model of melanoma, and characterization of their cell surface markers demonstrated co‐expression of CD11b, F4/80, and Stabilin‐1 (STAB1).^127^ Since this initial finding, additional cell surface markers that have been identified to co‐express with LYVE‐1 include FOLR2, podoplanin (PDPN), TIM4, and STAB1 (Table 1). These markers have emerged from both immunofluorescence and single‐cell studies.^5^, ^119^, ^127^, ^128^ Additional markers originating from single‐cell studies include GAS6, C1Q, and APOE.^5^, ^119^ An overview of studies that have identified and characterized this macrophage population using these various cell surface markers in mouse models and breast cancer tissue is found in Table 1. Collectively, these studies suggest that the TLF+ macrophages identified in normal tissues may also be involved in contributing to tumor progression.

**TABLE 1:** Macrophage heterogeneity in breast cancer. Macrophage subsets associated with LYVE‐1 have been identified in breast cancer in both human and mouse models with varied gene and protein expression. Including normal breast tissue, LYVE‐1, STAB1, TIM4, TREM2, FOLR2, and PDPN have all been found co‐expressed with LYVE‐1. In breast cancer, co‐expression of these proteins varies between different tumors.

Publication	LYVE1	STAB1	TIM4	TREM2	FOLR2	PDPN	Model	Function	Additional marker	Localization
Riabov et al. 2016^134^		**✓**					Human breast cancer, mouse	Pro‐tumor: efferocytic	NA	Not reported
Elder et al. 2018^48^						**✓**	Mouse	Immunosuppression, lymphoinvasion	NA	Near tumor lymphatics
Bieniasz‐Krzywiec et al. 2019^130^						**✓**	Human breast cancer and mouse 4 T1	Matrix remodeling, lymphangiogenesis and lymphoinvasion	NA	Near tumor lymphatics
Mutka et al. 2022^136^		**✓**					Human primary tumor	Intratumoral STAB1+: CD8+ T cell infiltration	NA	Intratumoral STAB1+: no change in DFS, improved DSS
								Peritumoral STAB1+: no reported function	NA	Peritumoral STAB1+: no change in DFS or DSS
Wang, Chaffee et al. 2020^6^	**✓**		**✓**		**✓**		Mouse 4 T1	Tissue remodeling	CD206, Gas6, Igf1, C1q	Peritumoral, resident
Ibrahim et al. 2020^128^	**✓**		**✓**				Mouse pre‐invasive lesion from PN1a BALB/c	Tissue homeostasis	Gas6	Stromal
				**✓**			Mouse pre‐invasive lesion from PN1a BALB/c	Lipid‐associated	Fabp5	Not reported
Opzoomer et al. 2021^131^	**✓**						MMTV‐PyMT	Proangiogenic	CD206	Perivascular
Timperi et al. 2022^152^	**✓**			**✓**	**✓**		Human breast cancer	Mo‐derived LAM‐STAB1: immunosuppressive	CD209, APOE	Localized to tumor stroma
		**✓**		**✓**	**✓**		Human breast cancer	LAM‐APOC1: inflammatory	C1q, APOE, Spp1	Not reported
Liu et al. 2022^154^				**✓**			Human breast cancer and mouse EMT6 model	Anti‐tumor	C1q, Spp1	Localized to tumor adipose
Nalio Ramos et al. 2022^119^				**✓**			Human breast cancer and MMTV‐PyMT mouse	Pro‐tumor	Spp1, APOE, C1q	Intratumoral (mouse)
	**✓**		**✓**		**✓**		Human breast cancer and MMTV‐PyMT mouse	Anti‐tumor: Positively associated with CD8+ T cell infiltration	Mrc1, APOE, C1q	Perivascular tumor stroma (human)

Lymphatic vessel endothelial hyaluronan receptor‐1+ macrophages exhibit varied functionality within tumors, including the promotion of tissue remodeling, immunosuppression, angiogenesis, and lymphangiogenesis. We have previously identified LYVE‐1+ macrophages in mammary glands of mice as well as in the peritumoral stroma of murine 4T1 mammary tumors.^6^ Furthermore, we have demonstrated that LYVE‐1+ macrophages in both the normal mammary gland and in the peritumoral stroma are selectively associated with hyaluronan‐associated regions and contribute to ECM remodeling.^6^

In addition to tissue remodeling, LYVE‐1+ macrophages, also marked by TIM4 and FOLR2, are also associated with immune regulation in the tumor microenvironment. In a human pan‐cancer analysis, Bugatti et al.^129^ identify two distinct TIM4+ macrophage subsets that are associated with differing effects on patient outcome. First, cavity TIM4 + FOLR2+ macrophages express immunosuppressive proteins such as triggering receptor on myeloid cells 2 (TREM2) and TGFβ and correlate with worse patient survival. Conversely, tertiary lymphoid structure TIM4 + FOLR2+ macrophage infiltration is associated with CD8+ T cell infiltration and correlates with better patient survival.^129^ In addition, FOLR2 + LYVE‐1 + TIM4+ macrophages have been found near blood vessels within human mammary tumors directly interacting with CD8+ T cells, resulting in a correlation between FOLR2+ macrophage count and patient survival.^119^ These data suggest that LYVE‐1 + FOLR2+ macrophages may exhibit distinct functions based on their spatial localization within the tumor.

In addition to the identification of LYVE‐1+ macrophages at the mammary tumor margin, LYVE‐1+ macrophages have also been associated with lymphatic vessels. For example, macrophages expressing PDPN, which is also co‐expressed with LYVE‐1 on macrophages, have been found in samples from breast cancer patients and are localized near tumor lymphatics.^130^ These PDPN+ macrophages bind galectin‐8 (GAL8) on lymphatic endothelial cells, which has been found to promote lymphatic endothelial cell migration. Further, PDPN+ macrophages are involved in extracellular matrix remodeling near lymphatics and promote lymphangiogenesis, and their deletion results in delayed breast cancer invasion. In another study, Elder et al.^48^ examined PDPN+ macrophages in tumors that form following orthotopic injection of cells into the mammary gland during involution to model post‐partum breast cancer. This study demonstrated that semaphorin 7A (SEMA7A) expression on tumor cells induces expression of PDPN on macrophages, which promotes migration of PDPN+ macrophages to lymphatic endothelial cell structures, thereby promoting lymphangiogenesis. In human breast cancer patients, co‐expression of SEMA7A, PDPN, and CD68 correlated with metastasis.

Similar to their localization in normal tissues, LYVE‐1+ macrophages have also been found in close association with blood vessels in tumors. For example, LYVE‐1+ macrophages were found to be localized near blood vessels in mammary tumors from a spontaneous model of mammary carcinoma, MMTV‐PyMT. These LYVE‐1+ macrophages were found to support perivascular mesenchymal cells and have a proangiogenic effect in tumors.^131^ LYVE‐1+ macrophages have also been found to respond to IL‐6, promoting the formation of cellular nests near blood vessels and ultimately leading to the exclusion of CD8+ T cells within tumors. When either IL‐6 or CCR5 are knocked out in MMTV‐PyMT mice, more CD8+ T cells are found within tumors and are more sensitive to chemotherapy compared to vehicle‐treated controls.^132^ This study highlights their spatial localization in relation to their angiogenic functionality.

A marker that co‐expresses with LYVE‐1 on macrophages, STAB1, is also known as CLEVER‐1 and functions as a scavenger receptor that binds and endocytoses acetylated low‐density lipoprotein (acLDL) and secreted protein acidic and rich in cysteine (SPARC).^133^ While STAB1 is found on sinusoidal endothelial cells, it is also expressed on LYVE‐1+ macrophages in tumors.^127^, ^133^ STAB1+ macrophages have been identified in both mouse models and human breast cancer patients, and injection of TS/A mammary adenocarcinoma cells into STAB1^−/−^ mice is associated with reduced tumor growth.^134^ STAB1+ macrophages have been functionally linked with phagocytic apoptotic clearance, an anti‐inflammatory, tumor‐supporting function in the TME.^50^, ^135^ STAB1+ macrophages have additionally been found in samples from human breast cancer patients, and the effects of STAB+ expression on patient outcome were stratified based on localization within the tumor.^136^ Intratumoral STAB1+ macrophage infiltration correlated with improved patient outcome and higher CD8+ T cell infiltration. However, no significant effects on patient outcome or immune cell infiltrate were correlated with STAB1+ macrophages localized to the peritumoral stroma.^136^ This study contradicts current literature regarding reported STAB1+ macrophage functionality; thus, more studies are needed to understand this macrophage subset.

Collectively, these studies demonstrate that macrophages expressing LYVE‐1 and associated markers (PDPN, TIM4, STAB1, and FOLR2) may have key roles in the context of breast cancer. These macrophages are found in various sites within the tumor including the peritumoral stroma, lymphatic vessels, blood vessels, and tertiary lymphoid structures. Emerging evidence supports the idea that spatial localization may contribute to regulating the functions of these macrophages, and the use of emerging spatial transcriptomic and proteomic technologies would be useful in further defining the environments of these distinct niches. Whether each of these subsets represent a unique macrophage subset with niche‐dependent functions warrants further investigation. In addition, further research needs to be done to parse the distinct functional differences between these subsets. For example, the potential relationship between these subsets and the TLF+ macrophages in normal tissues and whether they represent a single macrophage subset or diverge into multiple subsets during tumor progression would be an interesting focus for future investigation.

### 7.2 Lipid associated macrophages

As described above, recent single‐cell analyses have identified LAM populations in both the normal breast and in breast cancer samples. LAMs have been extensively characterized in atherosclerosis and other metabolic diseases such as obesity and tuberculosis.^137^, ^138^, ^139^ LAMs are characterized by a lipid‐related gene signature and have been reported in multiple cancer types such as breast cancer, gastric cancer, colorectal carcinoma, and pancreatic cancer.^110^, ^140^, ^141^, ^142^ Lipid metabolism in TAMs is associated with immunosuppression and resistance to chemotherapy, and lipid accumulation is associated with increased expression of PD‐L1 on TAMs in a gastric cancer model.^143^ Macrophages associated with lipid‐related genes including *Cd36* and *Plin2* have also been found in a high‐fat diet‐induced obesity model of breast cancer.^144^ These macrophages were found to promote mammary epithelial cell progenitor activity and increased DNA damage in p53‐deficient mammary epithelial cells, suggesting a role for in directly interacting with epithelial and tumor cells. Furthermore, a population of LAMs expressing TREM2 has been reported to have immunosuppressive functions, as have several other macrophages of tissue‐resident origin.^145^, ^146^

Triggering receptor on myeloid cells 2 is a type 1 transmembrane protein expressed on immune cells such as macrophages, dendritic cells, and microglia, as well as non‐immune cells such as osteoclasts.^147^ TREM2 binds lipids and glycosaminoglycans and can signal intracellularly through DNAX‐activating protein of 12 kD (DAP12).^148^ Activation of DAP12 can result in downstream signaling through PI3K, ERK, PLCy, and VAV, promoting cell survival and phagocytosis.^149^ In addition to its functions as a transmembrane protein, the soluble ectodomain of TREM2 can be cleaved by cell surface a disintegrin and metalloproteinase (ADAM) 10/17 resulting in a soluble TREM2 (sTREM2) fragment.^150^ sTREM signals through an unknown receptor to activate PI3K and NFκB and is thus associated with cell survival and inflammation.^150^ In microglia, TREM2 mediates the phagocytic uptake of apoptotic neurons and reduces subsequent inflammation.^151^

Lipid‐associated macrophages in both mouse and human adipose tissue express lipid‐associated genes such as *Lgals3*, *Fabp5*, and notably *TREM2*.^140^ Primary functions of TREM2+ LAMs include lipid uptake and tissue homeostasis, and genetic deletion of TREM2 leads to adipocyte hypertrophy and systemic hypercholesterolemia. In human adipose, the LAM gene signature is associated with phagocytosis and lipid metabolism. LAMs also stain positive for BODIPY, a stain used to identify lipid. Upon deletion of TREM2 in mice, adipose tissue macrophages show reduced BODIPY levels compared to wild‐type control mice. Further, upon TREM2 deletion, mice gain more weight and have more insulin, cholesterol, and LDL in their serum. These studies suggest that TREM2 is essential for LAM phenotype and function, although further studies are needed to define the exact mechanisms by which TREM2 mediates the LAM phenotype.

In human TNBC patients, high TREM2+ macrophage infiltration correlates with worse patient survival.^145^ In immune checkpoint blockade‐resistant human breast cancer patients, a population of monocyte‐derived STAB+TREM2+ LAMs is expanded, and this subset is associated with the tumor stroma.^152^ Further, cancer associated fibroblasts reprogram monocytes to the immunosuppressive LAMs phenotype, and depleting this population results in delayed tumor growth.^152^ Using scRNA‐seq analysis, we identified a population of LAMs in lung metastases of mammary tumor‐bearing mice, which were found in greater abundance than tumor‐free mice.^153^ Furthermore, we found that LAMs in metastatic lungs are positive for the alveolar macrophage marker Siglec‐F, suggesting that LAMs may arise from tissue‐resident macrophages. In addition, this LAM macrophage population has enriched gene sets associated with immunosuppression. Consistent with a tumor‐promoting role for LAMs in breast cancer, others have demonstrated that the depletion of LAMs with clodronate in an EMT6 model of murine breast cancer results in decreased tumor burden. Furthermore, LAMs have been identified in human breast cancer samples and are also positive for *Trem2*, *C1qc*, and *Spp1*.^154^ Additional scRNA‐seq data from human breast cancer identifies a monocyte‐derived macrophage population expressing *TREM2*, *SPP1*, *C1QA*, and *APOE*.^155^ Notably, this cluster also expresses *CD204*, which was previously demonstrated to be a prognostic factor by immunohistochemistry in breast cancer.^57^ Interestingly, both LYVE‐1+ and TREM2+ macrophages have been shown to express *C1q* genes (Table 1), raising the possibility that TREM2+ macrophages either overlap with or are derived from tissue‐resident LYVE‐1+ macrophages. Further studies defining the source of LAMs within the tumor microenvironment, the mechanisms by which macrophages accumulate lipid, and their spatial localization will provide important insights into this emerging tumor associated macrophage population.

## 8 TARGETING MACROPHAGE HETEROGENEITY IN CANCER

The significant roles of macrophages in regulating several aspects of tumor growth and progression make them an attractive target for cancer therapy.^156^ The balance between the tumor‐promoting and anti‐tumor properties of TAMs is an important consideration while investigating druggable targets and developing therapies. An ideal therapeutic strategy would involve inhibiting the tumor‐promoting effects of macrophages while simultaneously enhancing their antitumor functions. To that end, several attempts have been made to target TAMs in preclinical and clinical cancer models.^157^ Targeting macrophage recruitment via the CCL2‐CCR2 axis has been found to reduce infiltration and immunosuppression in breast, pancreatic, and hepatocellular carcinomas and synergize with cytotoxic T cell activation.^105^, ^158^, ^159^ Re‐directing TAMs to a pro‐inflammatory, anti‐tumor phenotype is being investigated using inhibitors of pathways such as phosphoinositide‐3‐kinase‐γ (PI3K‐γ), which have reported increased expression of pro‐inflammatory cytokines such as TNF‐α, IL‐12, and MHCII and decreased expression of immunosuppressive factors such as TGF‐β, IL‐10, CCL2, and ARG1.^157^ Inhibition of PI3Kγ significantly reduces tumor growth and metastasis in synergy with cytotoxic T cell infiltration.^160^ Several other strategies are being investigated in clinical and preclinical models to achieve re‐polarization of macrophages, such as targeting receptor‐protein kinase 1 (RIPK1), activating toll‐like receptor (TLR) with agonists, and targeting the complement pathway among others.^157^ Inhibiting macrophage proliferation and survival has been extensively investigated as a strategy for targeting macrophages, specifically through inhibition of CSF1R.^161^ Antibody‐mediated targeting of the CSF1‐CSF1R axis and depletion of macrophages through pharmacological CSF1 inhibition demonstrated reduced tumor progression and increased chemosensitivity in several mouse models of cancer.^162^, ^163^, ^164^ In human trials, inhibiting CSF1R in patients has had limited effect; however, it may be effective in combination with immunotherapy.^165^

While targeting macrophages for treatment of solid tumors presents a promising therapeutic strategy, the use of pan‐macrophage inhibitors has proven to be challenging due to issues with side effects and low efficacy in clinical trials.^166^ Macrophages are highly heterogeneous and have both pro‐ and anti‐tumor functionality; thus, understanding macrophage heterogeneity and context‐specific effects of TAMs may lead to improved breast cancer therapies.

Given the extensive studies focused on LYVE‐1+ macrophages, targeting LYVE‐1 has been investigated in tumor models. A rat monoclonal antibody has been developed that targets the extracellular domain of LYVE‐1.^167^ This antibody was designed to target lymphatic endothelial cells and has been shown to reduce lymphatic endothelial migration and tube formation. In addition, nude mice that were intradermally injected with MDA‐MB‐231 breast cancer cells and treated with the LYVE‐1 monoclonal antibody demonstrated reduced primary tumor growth with the caveat that this model lacks T cells and thus does not recapitulate the tumor immune microenvironment. Macrophages were not examined in this model; however, further investigation is warranted to determine whether this LYVE‐1 monoclonal antibody may be a viable method to target LYVE‐1+ macrophages.

While LYVE‐1+ macrophages have been widely reported to have pro‐tumor properties and make a tempting target for clinical inhibition, the expression of LYVE‐1 is not restricted to macrophages. LYVE‐1, STAB1, and PDPN are also expressed on lymphatic endothelial cells, and targeting cells expressing these proteins would most likely have unwanted off‐target effects.^168^ Also expressed on LYVE‐1+ macrophages, FOLR2 may be a druggable target for the inhibition of LYVE‐1+ macrophages, as FOLR2 expression is more restricted to macrophages.^169^, ^170^, ^171^ Notably, targeting FOLR2 with a monoclonal antibody reduced tumor growth in a humanized mouse model of acute myeloid leukemia, coupled with NK cell transfer.^172^ Further studies are needed to determine whether FOLR2 may be a druggable target for breast cancer patients.

Triggering receptor on myeloid cells 2+ macrophages are also emerging as a potential clinical target. Using a monoclonal antibody to target TREM2+ macrophages, Binnewies et al.^173^ reported a decrease in tumor growth in an orthotopic murine model of ovarian cancer, which was further enhanced when combined with anti‐PD‐1 therapy. Further investigation into TREM2 as a potential clinical target has culminated in a clinical trial testing the efficacy of a humanized TREM2 targeting antibody in unresectable or metastatic breast, colorectal, lung, renal, and ovarian tumors after relapse following standard of care treatment.^174^ The treatment has also been approved as combination therapy with the PD‐1‐targeting monoclonal antibody pembrolizumab; however, results have not been reported. These examples demonstrate that efforts to understand macrophage heterogeneity with normal tissue and tumors have led to new and promising macrophage targeting approaches.

## 9 CONCLUSIONS AND FUTURE DIRECTIONS

Recent studies have highlighted the phenotypic and functional heterogeneity of macrophages in normal mammary tissue as well mammary tumors. Several emerging studies have suggested the potential importance of the spatial localization of macrophages within tissues for their phenotype and function. Thus, further investigation using emerging spatial technologies is required to understand how the microanatomical niche contributes to macrophage heterogeneity in these tissues. In addition, further research is needed to understand how macrophages within these distinct spatial regions interact with other stromal populations as well as other cell types of the immune system. While there are clear parallels between tissue resident macrophages in the normal mammary gland in breast cancer, further investigation is needed to define the specific contributions of resident macrophages versus infiltrating macrophages to TAM subsets in cancer and the relationship with the extensive heterogeneity observed in the TME. While macrophage heterogeneity has been identified in both mouse models and human breast cancer samples, additional research is needed to fully understand the significance of macrophage heterogeneity within breast cancer subtypes. As therapies directed at specific macrophage subsets emerge, such as TREM2‐based therapies, identifying the presence of these macrophage subsets across the breast cancer subtypes will be important for developing the most effective therapeutic strategies. Finally, further investigation into how the activation and expansion of anti‐tumor macrophage subsets may be harnessed in the clinic may lead to novel therapeutic approaches that engage anti‐tumor immune responses. For example, advances in cell‐based immune therapies such as emerging chimeric antigen receptor (CAR)‐macrophages as well as antibody‐based therapies may be capable of delivering stimulating factors and thereby improving anti‐tumor function.^175^

Macrophages are being increasingly recognized as one of the significant players in tumor progression, metastasis, and response to targeted therapy. Deeper understanding into the transcriptomic landscape of TAMs has pivoted our understanding away from the approach of viewing macrophages as either M1 or M2 polarized and has highlighted the various subsets within the tumor that exist on a spectrum. Identifying tumor‐promoting macrophage populations and finding ways to effectively target such subsets will help us develop effective therapies targeting the stroma. Concurrently, increasing the anti‐tumor properties of inhibitory macrophage populations will also be crucial in utilizing this versatile, abundant, and plastic cell type to its full potential.

## AUTHOR CONTRIBUTIONS

**Alexis K. Elfstrum:** Conceptualization (equal); writing – original draft (equal); writing – review and editing (equal). **Aditi S. Bapat:** Conceptualization (equal); writing – original draft (equal); writing – review and editing (equal). **Kathryn L. Schwertfeger:** Conceptualization (lead); funding acquisition (lead); writing – original draft (lead); writing – review and editing (lead).

## FUNDING INFORMATION

Funding provided by National Institutes of Health, National Cancer Institute (R01CA265004, R01CA215052) and National Institutes of Health, National Institute of Child Health and Development (R01HD95858, R01HD106929) to (KLS).

## CONFLICT OF INTEREST STATEMENT

The authors state that they have no conflicts of interest.

## Data Availability

Not Applicable.
